# Steroid-Responsive Involuntary Movements as a Remote Symptom of Febrile Infection-Related Epilepsy Syndrome

**DOI:** 10.7759/cureus.60525

**Published:** 2024-05-17

**Authors:** Ayaka Ohno, Shimpei Baba, Wataru Jinnnai, Hiroki Hoshino, Hideaki Kanemura, Takashi Saito, Yuko Shimizu-Motohashi, Hirofumi Komaki

**Affiliations:** 1 Department of Child Neurology, National Center Hospital, National Center of Neurology and Psychiatry, Tokyo, JPN; 2 Department of Pediatrics, Toho University Medical Center Sakura Hospital, Chiba, JPN

**Keywords:** autoimmune encephalopathy, involuntary movements, autoimmune chorea, chronic phase, febrile infection-related epilepsy syndrome (fires)

## Abstract

Febrile infection-related epilepsy syndrome (FIRES) is a rare epileptic encephalopathy that occurs in children or adolescents. To date, evidence for the management of the post-acute phase of FIRES is focused on drug-resistant epilepsy that continues from the acute phase. Information on involuntary movements, which are newly developed in the chronic phase, is limited. We report a 13-year-old boy, who had a history of FIRES at nine years of age and experienced worsening seizure control that was accompanied by unremitting involuntary　movements after two years of a fairly controlled period. The involuntary movements resulted in motor deterioration and forced him to be bedridden. Although no neuronal autoantibodies were detected, we hypothesized that the boy’s neurological deterioration was triggered by an autoimmune response based on the elevation of serum anti-glutamic acid decarboxylase and serum anti-thyroid peroxidase antibodies and hypermetabolism of bilateral lenticular nuclei on 18-fluorodeoxyglucose positron emission tomography that resembled those reported in patients with other types of autoimmune encephalitis. Serial methylprednisolone pulse therapy and intravenous immunoglobulin therapy ameliorated involuntary movements and improved his activities of daily living. Late-onset involuntary movements, along with seizure exacerbation, may appear in the chronic phase of FIRES. Immunotherapy could be effective in treating these symptoms.

## Introduction

Febrile infection-related epilepsy syndrome (FIRES) is an epileptic encephalopathy that develops after a prior febrile infection, with fever starting between two weeks and 24 hours before the onset of super-refractory status epilepticus (SRSE), with or without fever at the onset of it [1]. Chronic refractory epilepsy and moderate to severe cognitive impairment, which follow without an intervening silent period, are known as chronic symptoms [2]. The types of seizures in the chronic phase are similar to those in the acute phase [3]. In many cases, epileptic seizures last a lifetime, but the frequency of seizures gradually decreases [4], and patients rarely become seizure-free [5]. Although several reports have described the manifestations and management of FIRES, reports of late-onset neurological deterioration, particularly involuntary movements, are scarce. The exact pathophysiology of FIRES is also yet to be elucidated. Herein, we report a case of a patient with a history of FIRES who presented with involuntary movements and exacerbation of seizures in the chronic phase, which was ameliorated by the administration of immunotherapy.

## Case presentation

The patient was a 13-year-old boy with normal development and was previously healthy. When he was nine years old, six days after a febrile episode, he developed SRSE and was diagnosed with FIRES. He required continuous thiopental infusion, immunosuppressive therapy including intravenous methylprednisolone pulse therapy (IVMP) and intravenous immunoglobulin therapy (IVIg), and one month of ventilator management. His motor and intellectual function severely deteriorated at the time of hospital discharge (e.g., he was bedridden, on tube feeding, and could not speak). Daily focal onset seizures persisted, but his overall condition was fairly controlled under multiple antiseizure medications in the following two years, with some recovery in his function (e.g., he could feed himself, run, and communicate with others using single words). From 11 years, however, his seizure control was exacerbated, and he exhibited generalized seizures 20-30 times a day. Moreover, from 12 years, the boy became bedridden concurrently with the development of involuntary movements. The involuntary movements disappeared for a month along with the seizures after administration of potassium bromide (Video 1, Movie 1) but flared up and could not be controlled thereafter.

**Figure 1:**
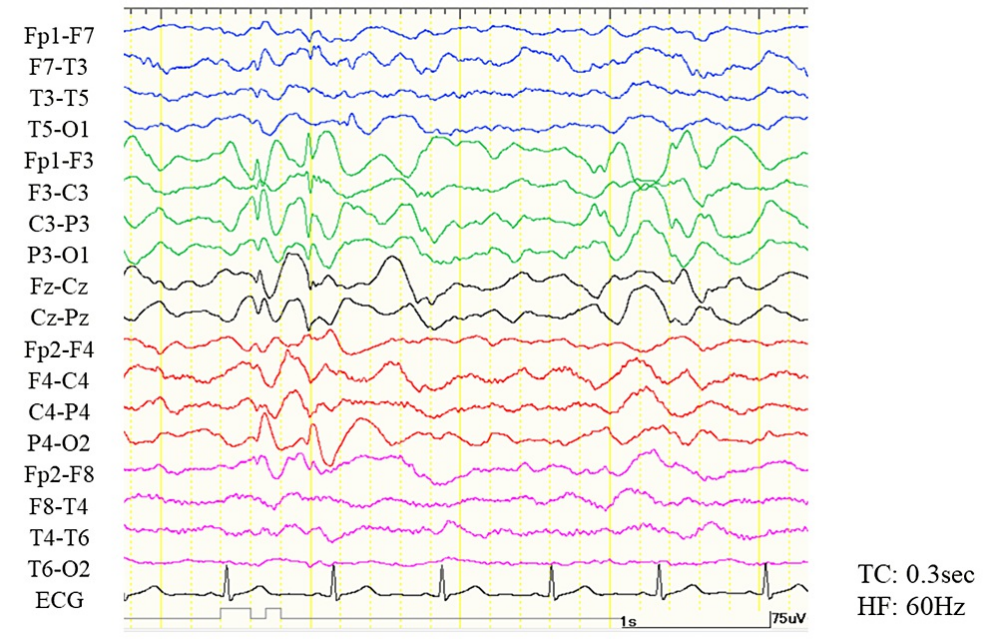
Sleep electroencephalogram before the initiation of immunotherapy. The bipolar montage shows continuous, generalized high-amplitude slow waves with/without spikes, predominantly seen in the left parasagittal region. HF, high-cut filter; TC, time constants.

At 13 years of age, the patient was transferred to our hospital for close examination. He could hold eye contact with others and had an interest in his surroundings, but he rarely spoke a word. Although his muscle tone was maintained, he could not hold a sitting position. Irregular, continuous, and massive movements observed in his face and extremities severely disturbed his activities of daily living (ADL), which we diagnosed as chorea (Video 1, Movie 2). Generalized onset tonic seizures were observed 30-40 times a day. Examination of cerebrospinal fluid (CSF) protein revealed mild protein elevation at 57 mg/dl, negative oligoclonal bands, and a normal IgG index at 0.44. On video-electroencephalogram monitoring, generalized high-amplitude slow waves with/without spikes were captured (Figure 1).

**Figure 2:**
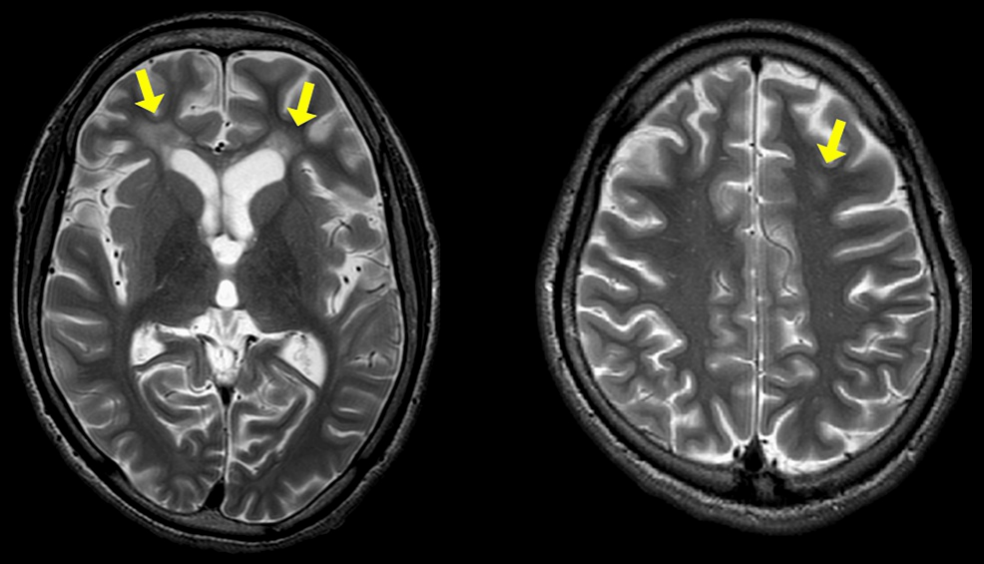
Magnetic resonance imaging findings before the initiation of immunotherapy. On T2-weighted images, diffuse cerebral atrophy and hyperintense lesions (arrows) in the periventricular white matter are observed.

**Figure 3:**
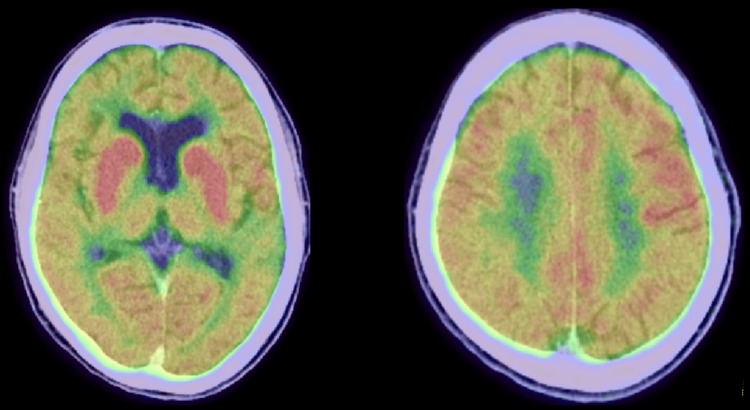
18-Fluorodeoxyglucose positron emission tomography before the initiation of immunotherapy. Strong hypermetabolism in the bilateral basal ganglia and diffuse cortical hypometabolism are observed.

As we could not observe any change in his electroencephalogram during the involuntary movements, we assumed the movements were nonepileptic. Brain magnetic resonance imaging (MRI) revealed T2 hyperintensity lesions in the periventricular white matter and diffuse cerebral atrophy (Figure 2), which remained unchanged from those recorded three years before. The 18-fluorodeoxyglucose positron emission tomography (FDG-PET) showed diffuse cortical hypometabolism and strong hypermetabolism in the bilateral basal ganglia (Figure 3).

On screening for neuronal autoantibodies, we confirmed a slight elevation of serum anti-glutamic acid decarboxylase (GAD) antibodies at 10.5 U/ml (normal range: < 5.0 U/ml) and serum anti-thyroid peroxidase (TPO) antibodies at 6.0 U/ml (normal range: < 3.3 U/ml), respectively; however, we failed to find a pathological elevation of those antibodies in the CSF. Other pathological neuronal autoantibodies against N-methyl-d-aspartate receptor, leucine-rich-glioma-inactivated 1 (LGI1) protein, contactin-associated protein-like 2, alpha-amino-3-hydroxy-5-methyl-4-isoxazole propionic acid (AMPA)-type glutamate receptor, gamma-aminobutyric acid type B, dipeptidyl-peptidase-like protein-6, amphiphysin, collapsin response mediator protein 5 (CV2), paraneoplastic antigen MA2, Ri, Yo, Hu, recoverin, sex-determining region Y protein (SRY)-related high-mobility group protein (HMG)-box gene 1, titin, zinc finger protein of the cerebellum 4 (zic4), glutamic acid decarboxylase 65, and delta/notch-like epidermal growth factor-related receptor (Tr) were all negative in the CSF.

**Figure 4:**
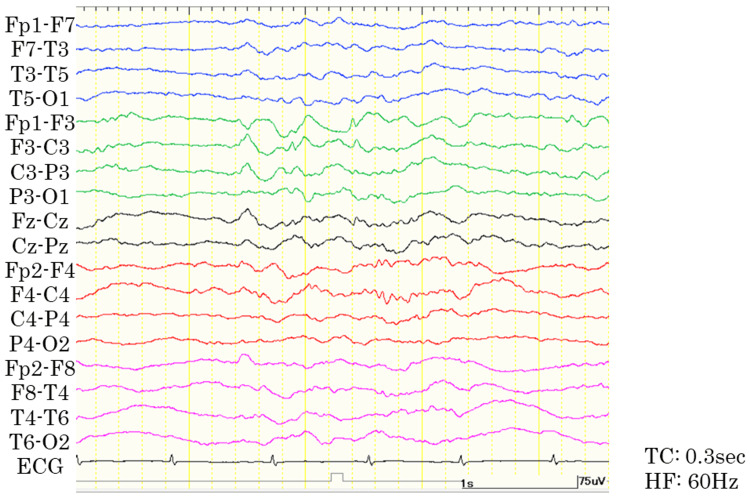
Sleep electroencephalogram nine months after the start of immunotherapy. The bipolar montage shows improvement in generalized slow waves, whereas frequent epileptiform discharges persist predominantly in the left frontal region. HF, high-cut filter; TC, time constants.

Based on the findings of FDG-PET and elevation of serum anti-GAD and anti-TPO antibodies, we hypothesized that an immunological response might trigger the development of chorea. We administrated immunotherapy including IVMP (1,000 mg for three consecutive days, repeated for two weeks) and subsequent oral prednisolone, IVIg, and mycophenolate mofetil. Around one week after the first IVMP, the chorea gradually improved. The seizures decreased from 20 to 30 per day to less than 10 per day and transformed from generalized onset tonic seizures to focal onset impaired awareness seizures. Electroencephalography findings also improved compared to those recorded at admission, showing improvement in generalized slow waves, whereas frequent epileptiform discharges persisted in the left frontal region (Figure 4).

Transient exacerbation of the chorea and seizure control was observed when the boy developed pneumonia and cellulitis (Video 1, Movie 3). One year after immunotherapy initiation, he could sit alone, walk with support, and play catch (Video 1, Movie 4). The results for serum anti-GAD antibodies and anti-TPO antibodies became negative. However, as the chorea flared up approximately three weeks after each IVMP, monthly IVMP administration was necessitated to maintain his ADL.

**Video 1:** Clinical course of the involuntary movements. Movie 1: At 12 years of age, five months before hospitalization. The boy could play at the batting center. Movie 2: The patient was admitted to our hospital at 13 years of age. The boy was bedridden because of chorea. Chorea was predominantly observed in the distal extremities and face. Movie 3: One week after the development of cellulitis, chorea worsened. The boy again had difficulty maintaining a sitting position. The patient’s symptoms improved after intravenous methylprednisolone pulse therapy (IVMP) administration. Movie 4: One year after initiation of immunotherapy, the boy was able to sit, walk short distances with assistance, and play catch. FIRES: febrile infection-related epilepsy syndrome.

## Discussion

Herein, we described the clinical course of involuntary movements of a patient with FIRES in detail. According to the recent diagnostic criteria [1,6], we assumed that the clinical course of the acute phase of our patient was consistent with the diagnosis of FIRES. However, there is minimal information about the presentation of involuntary movements along with the deterioration of ADL in the literature on post-acute FIRES. At first, we suspected that the boy co-developed some other autoimmune encephalitis; as our thorough search for pathogenic neuronal autoantibodies failed, we determined that we faced an unreported aspect of chronic FIRES. A case series reported that the motor function of patients with FIRES declines after the acute phase and improves in the long-term follow-up [5]. The clinical course of our case was similar to that of the patients in the study up to the first two years of the chronic phase; our patient newly developed involuntary movements with a decline in motor/intellectual function. The boy’s clinical course implies the pathophysiology of FIRES exhibits a potentially progressive nature even in the chronic phase, not only the drastic deterioration seen in the acute phase. We believe that our case is informative for clinicians who encounter patients with FIRES.

Although the pathophysiology of FIRES has not been clarified to date, recent evidence indicates that non-antibody-mediated neuroinflammation is related to the development of FIRES [2,4]. This involves an imbalance between pro- and anti-inflammatory mediators, causing activation of innate immune pathways in multiple cell types and resulting in an uncontrolled neuroinflammatory cascade [7]. Our case indicates that the neuroinflammation process may not end in the acute phase but may continue playing an important role in forming the chronic phase due to the following factors. First, the strong hypermetabolism in the bilateral basal ganglia in the FDG-PET is similar to those in patients with autoimmune encephalitis related to anti-LGI1 antibody [8,9]. Second, mild elevation of anti-GAD and anti-TPO antibodies in the serum is unlikely to be the cause of the disease but was considered a marker of autoimmunity [10]. Third, immunotherapy was effective, albeit partially, in controlling seizures/involuntary movements. Clarifying the detailed mechanisms of autoimmunity is necessary to establish a better treatment strategy for the chronic phase of FIRES.

## Conclusions

We reported a 13-year-old boy who presented involuntary movements and worsening seizure control in the chronic period of FIRES. In the chronic phase of FIRES, involuntary movements can occur as remote symptoms along with seizure exacerbation, even after a reasonably stable period. Immunotherapy may alleviate these symptoms. Currently, the pathomechanism of the chronic phase of FIRES has not been elucidated; our case suggests that the neuroinflammation process, believed to play an essential role in forming the acute phase of FIRES, may continue in its chronic phase. We believe our experience will help clinicians who care about the long-term health status of patients with FIRES.
